# m^6^A mRNA methylation regulates CTNNB1 to promote the proliferation of hepatoblastoma

**DOI:** 10.1186/s12943-019-1119-7

**Published:** 2019-12-23

**Authors:** Li Liu, Jing Wang, Guifeng Sun, Qiong Wu, Ji Ma, Xin Zhang, Nan Huang, Zhixuan Bian, Song Gu, Min Xu, Minzhi Yin, Fenyong Sun, Qiuhui Pan

**Affiliations:** 1 Department of Clinical Laboratory Medicine, Shanghai Children’s Medical Center, School of medicine, Shanghai Jiaotong University, Shanghai, 200127 China; 2 Department of Clinical Laboratory, Shanghai Fourth People’s Hospital affiliated to Tongji University School of Medicine, Shanghai, 200081 China; 3 Department of Surgery, Shanghai Children’s Medical Center, School of medicine, Shanghai Jiaotong University, Shanghai, 200127 China; 4 Department of Clinical Laboratory Medicine, Shanghai Tenth People’s Hospital of Tongji University, Shanghai, 200072 China; 5 School of Pharmacy, Guangdong Pharmaceutical University, Gaungzhou, 510006 China; 6 Department of Pathology, Shanghai Children’s Medical Center, School of medicine, Shanghai Jiaotong University, Shanghai, 200127 China

**Keywords:** RNA m^6^A methylation, Wnt/β-catenin pathway, CTNNB1, Hepatoblastoma, METTL3

## Abstract

**Background:**

N^6^-Methyladenosine (m^6^A) modification has been implicated in many biological processes. It is important for the regulation of messenger RNA (mRNA) stability, splicing, and translation. However, its role in cancer has not been studied in detail. Here we investigated the biological role and underlying mechanism of m^6^A modification in hepatoblastoma (HB).

**Methods:**

We used Reverse transcription quantitative real-time PCR (RT-qPCR) and Western blotting to determine the expression of m^6^A related factors. And we clarified the effects of these factors on HB cells using cell proliferation assay, colony formation, apoptotic assay. Then we investigated of methyltransferase-like 13 (METTL3) and its correlation with clinicopathological features and used xenograft experiment to check METTL3 effect in vivo. m^6^A-Seq was used to profiled m^6^A transcriptome-wide in hepatoblastoma tumor tissue and normal tissue. Finally, methylated RNA immunoprecipitation (MeRIP) assay, RNA remaining assay to perform the regulator mechanism of MEETL3 on the target CTNNB1 in HB.

**Results:**

In this research, we discovered that m^6^A modifications are increased in hepatoblastoma, and METTL3 is the main factor involved with aberrant m^6^A modification. We also profiled m^6^A across the whole transcriptome in hepatoblastoma tumor tissues and normal tissues. Our findings suggest that m^6^A is highly expressed in hepatoblastoma tumors. Also, m^6^A is enriched not only around the stop codon, but also around the coding sequence (CDS) region. Gene ontology analysis indicates that m^6^A mRNA methylation contributes significantly to regulate the Wnt/β-catenin pathway. Reduced m^6^A methylation can lead to a decrease in expression and stability of the CTNNB1.

**Conclusion:**

Overall our findings suggest enhanced m^6^A mRNA methylation as an oncogenic mechanism in hepatoblastoma, METTL3 is significantly up-regulated in HB and promotes HB development. And identify CTNNB1 as a regulator of METTL3 guided m^6^A modification in HB.

## Introduction

Hepatoblastoma (HB) is the most common pediatric liver cancer. It is an embryonal neoplasms and mostly can be diagnosed during the first three years of life. It originates from undifferentiated hepatic progenitor cells, and undergo a malignant transformation during embryogenesis [1, 2]. Typical therapeutic strategies such as combined surgery and chemotherapy have demonstrated improved outcomes for children with HB. However, the prognosis for patients with advanced or chemotherapy-refractory disease is still very poor [3–5]. Components of the Wnt/β-catenin pathway are frequently mutated and overactive in solid malignancies and promote tumor development [6]. In case of HB, CTNNB1 encoding β-catenin, is the most recurrently mutated driving proto-oncogene gene with 50–90% frequency. The point mutations and in-frame deletion of the exon3 in CTNNB1, has been reported as the primary cause of HB. In-frame deletions or missense mutations within exon 3 are gain-of-function mutations can lead to a degradation-resistant β-catenin protein that accumulates in the nucleus, binds to the TCF4/LEF − 1 transcription factor, and drives the activation of target genes such as Jun, c-Myc and Cyclin D1 [7–9]. Moreover, products of the loss-of-function somatic mutations of tumor suppressor genes AXIN1 and AXIN2 affecting the β-catenin degradation have also been reported in HB [10].

m^6^A is considered as the most common internal modification in messenger RNAs (mRNAs). These modifications are generally present on adenosine embedded in the consensus sequence G [G > A]m6 AC[U > A > C] across the mRNA transcripts, especially near the stop codons [11]. Recent studies have demonstrated that m^6^A modifications in mRNAs or non-coding RNAs can influence RNA fate and functions such as mRNA stability, splicing, transport, localization and translation, primary microRNA processing, and RNA-protein interactions [12–14]. Also, m^6^A modifications are critical for most bioprocesses including tissue development, Deoxyribonucleic acid (DNA) damage response, sex determination, and tumorigenesis [11, 15, 16]. The reversible process of m^6^A formation occurs through a multicomponent methyltransferase complex consisting of the methyltransferase-like 13 (METTL3)/methyltransferase like 14 (METTL14) heterodimeric catalytic core and a regulatory subunit, Wilms’ tumor 1-associating protein (WTAP), KIAA1429, RNA Binding Motif Protein 15 (RBM15) and Zinc finger CCCH domain-containing protein 13 (ZC3H13), [17, 18]. METTL3, METTL14, and WTAP constitute the core of the methyltransferase complex. METTL3 possesses an active methyltransferase domain that catalyzes the conversion of adenosine (A) to m^6^A, METTL14 acts as the RNA-binding platform, and therefore, the METTL3-METTL14 forms a stable heterodimer and highly conserved in mammals. The heterodimer is required for the methylation activity, WTAP interacts with METTL3 and METTL14 to modulate levels of RNA transcripts [19–22]. The reversible process is conducted by m^6^A eraser fat mass and obesity-associated protein (FTO) and alkB homolog 5 (ALKBH5). The FTO and ALKBH5 serve as two mammalian RNA demethylases to reverse the m^6^A modifications [23–25]. m^6^A modification needs to be recognized by reader proteins in order to exert their biological functions. YT521-B homology (YTH) domain family proteins are reported to be m^6^A readers. Among these, YTHDF1 as the reader, located at the 5′ untranslated region of m^6^A has been suggested to promote mRNA translation efficiency in a cap-independent manner [26]. Another m^6^A reader YTHDF2 binds to m^6^A at the 3′ untranslated region through its C-terminal YTD domain and localizes the targeted mRNA to processing, bodies for accelerated degradation through its N-terminal domain [12]. Furthermore, heterogeneous nuclear ribonucleoprotein (hnRNP) could also serve as a potential nuclear m^6^A reader that can influence mRNA localization and alternative splicing [11]. Given the crucial role of m^6^A modification in regulating gene expression and various biological processes, it is reasonable to speculate that aberrant m^6^A modification might also be involved in carcinogenesis. Previous research had shown that m^6^A eraser protein ALKBH5 has the function of regulating cancer stem cell properties [27]. FTO has been reported to serve as an oncogene in acute myeloid leukemia [28]. METTL3 depletion can leads to apoptosis through the p53 signaling, also it can promote liver cancer (HCC) progression through YTHDF2 dependent post-transcriptional silencing of suppressor of cytokine signaling 2 (SOCS2), [29, 30]. However, research on the role of METTL3 and the m^6^A machinery in cancers including hepatoblastoma still in its early stage, indicating an urgent need to head light on future clinical cancer therapy and drug developments.

In the present study we assessed levels of m^6^A, its key catalytic genes and profiled m^6^A transcriptome-wide in HB tissues. The level of m^6^A was increased in HB, and METTL3 was found to be the main factor involved in aberrant m^6^A modification. Furthermore, we also explored the target gene CTNNB1 and addressed the mechanism of methyltransferase METTL3 participates modulate CTNNB1 in HB.

## Materials and methods

### HB specimens and cell lines

Primary HB tumors and their nontumor counterparts from 25 patients were used. The study was approved by the Shanghai Children’s Medical Center affiliated to Shanghai Jiaotong University School of Medicine Institutional Review Board. Informed consent was obtained from each participant. Human HB cell line Huh6 was purchased from Cell Bank of Type Culture Collection of the Chinese Academy of Sciences (Shanghai, China). Human HB cell line HepG2, human normal liver cell lines (QSG-7701, HL-7702) and HEK293T were purchased from American Type Culture Collection (ATCC) (Maryland, USA). HepG2 cells were cultured in Minimum Essential Medium (MEM) (Gibco, USA) supplemented with 10% (v/v) Fetal Bovine Serum (FBS) (Gibco, USA) and 1% (v/v) antibiotics (penicillin streptomycin) (Gibco, USA), Huh6, HEK293T and HL-7702 cells were cultured in Dulbecco’s Modified Eagle’s Medium (DMEM) (Gibco, USA) with 10% Fetal Bovine Serum (FBS) (Gibco, USA) and 1% (v/v) antibiotics (penicillin streptomycin) (Gibco, USA), QSG-7701 cells were cultured in RPMI-1640 Medium (Gibco, USA) supplemented with 10% (v/v) Fetal Bovin Serum (FBS) (Gibco, USA) and 1% (v/v) antibiotics (penicillin streptomycin) (Gibco, USA). Cells were cultured at 37 °C in an atmosphere with 5% CO_2_.

### Measurement of m^6^A level

m^6^A level in total RNA were detected using a commercial m^6^A RNA methylation quantification kit (Abcam, UK). Briefly, 200 ng total RNA was added into each well and the capture antibody solution and detection antibody solution were added according to the manufacturer’s protocol. The absorbance of each well at a wavelength of 450 nm was colorimetrically measured for m^6^A level.

### High throughput m^6^A sequencing

Total RNA was extracted from HB tumor and normal tissues. RNA was tested for quality using nanodrop and gel electrophoresis. RNA was randomly fragmented to ~ 200 nucleotides by RNA fragmentation reagents. Fragmented RNA was incubated for 2 h at 4 C with m^6^A antibody (Synaptic System, Cat. No. 202003, diluted to 0.5 μg/ul) for immunoprecipitation following the standard protocol of the Magna methylated RNA immune precipitation (MeRIP) m^6^A kit (Merk Millipore, MA). The mixture was then incubated with beads and eluted with elution buffer (1 × IP buffer and 6.7 mM m^6^A). Eluted RNA was precipitated by 75% ethanol. The eluted RNA was treated with RNasin (Ambion, Cat No. AM2694) according to the manufacturer’s instructions. TruSeq Stranded mRNA Sample Pre Kit (Illumina) was used to construct the library from immunoprecipitated RNA and input RNA following to a published protocol. Sequencing was performed on an Illumina HiSeq machine with 2 × 100 cycles Solexa paired-end sequencing.

### RNA extraction and quantitative PCR (qPCR) analysis

Total RNA was isolated using TRIzol reagent (Invitrogen, USA) following the manufacturer’s protocol and quantified by nanodrop 2000. Briefly, cells were lysed with TRIzol reagent at room temperature for more than 5 min and chloroform was added. After centrifugation at 12,000 × rpm for 15 min the aqueous phase was collected and mixed with isopropanol before centrifuging at 12,000 × rpm for 10 min at 4 °C. RNA was dissolved in RNase-free water and recovered by centrifuge. For analysis of mRNA expression, 200–500 ng of RNA was converted to cDNA using PrimeScript™ RT Regent Kit (TAKARA, Dalian, China). Quantitative real-time PCR using KAPA SYBR® FAST qPCR Kit Master Mix (2×) Universal (Applied Biosystems, USA) was performed on a 7500 Fast Real-time PCR system (Applied Biosystems). Total RNA was isolated for qPCR analysis. Quantitative PCR primers sequence are listed in Additional file 3: Table S1. GAPDH was used as a normalizing gene in all experiments.

### Western blot assay

Equal amounts (around 0.2 g) of tissues and counted cells were homogenized in RIPA buffer (0.1 g/mL) (Beyotime, China) by using a Speed-Mill PLUS homogenizer (Analytik Jena, Jena, Germany). The homogenate was then centrifuged at 12,000 × rpm for 20 mins at 4 °C, and the supernatant proteins were collected. After the measurement of protein concentration, the proteins were boiled in 1× sodium dodecyl sulfate (SDS) sample buffer (100 μl for 1× 10^6^ cells) for 5 mins, and then equal amounts of protein (50 μg) were loaded and separated by 10% sodium dodecyl sulfate-polyacrylamide gel electrophoresis (SDS-PAGE) gel and transferred to polyvinylidene fluoride (PVDF) membrane (GE Healthcare, Buckinghamshire, UK). The membrane was then incubated with specific primary antibodies (Additional file 4: Table S2) and fluorescently conjugated secondary antibodies (Licor, USA), followed by the detection with the Odyssey system (TAITEC Co., Saitama, Japan).

### Cell proliferation assay

The cell proliferation was assessed by CCK-8 (Beyotime, China) following the manufacturer’s instructions. Briefly, cells were seeded on a 96-well plate in triplicates at a density of 1000 cells/200 μl. The medium in each well was replaced with 100 μl of fresh medium supplemented with 10 μl of CCK-8 reagent. At indicated time points and incubated at 37 °C for 3–4 h. The optical density (absorbance) at a wavelength of 450 nm was determined by a multiplate reader (Bio Tek, Vermont, USA).

### Colony-formation assay

Cells were plated into 12-well plates at a density of 1000 cells. After incubation for approximately 7 days, the cells in 12-well plates were fixed with 4% paraformaldehyde (PFA) and stained with 1% crystal violet solution to visualize their colony-forming ability.

### Apoptosis assay

For apoptosis assays, FITC Annexin V apoptosis Detection Kit (BD Biosciences, San Diego, CA) was used following the manufacturer’s specifications. Briefly, the hepatoblastoma cells (2 × 10^5^ cells/plate) in 6-well plates cells were incubated for 48 h. Cells were collected by mild trypsinization washed twice with cold PBS, stained with FITC-Annexin V and propidium iodide (PI) on ice for 5 mins, and subjected to flow cytometric analysis using a BD LSRFortessa analyzer (BD Biosciences).

### Quantification of mRNA methylation with m^6^A-IP and RT-qPCR

m^6^A-IP enrichment followed by RT-qPCR to quantify the changes in m^6^A methylation of the target gene was performed using Magna MeRIP m^6^A Kit (Millipore, MA) following the manufacturer’s instructions. 5 μg of fragmented mRNA extracted from HepG2 or Huh6 stable cells was incubated with 5 μg m^6^A antibody (202,003, Synaptic Systems) or mouse IgG (CS200621, Millipore)-conjugated beads in 500 μl 1 × IP buffer for 4 h at 4 °C. Methylated RNA was eluted by free m^6^A from the beads and purified with RNeasy Mini kit (217,004, Qiagen). One tenth of the fragmented RNA was saved as an input control for standardization. The relevant enrichment of m^6^A of CTNNB1 in each sample was analyzed by RT-qPCR.

### LC-MS/MS quantification of m^6^A in total RNA

Total RNA was isolated using TRIzol reagent (Invitrogen, USA) following to the manufacturer’s instruction. 1 μg of total RNA was digested by 4 μl nuclease P1 (Sigma, USA) in 40 μl buffer solution (10 mM Tris-HCl pH 7.0, 100 mM NaCl, 2.5 mM ZnCl2) at 37 °C for 12 h, followed by incubating with 1 μl alkaline phosphatase (Sigma, USA) at 37 °C for 2 h. RNA solution was diluted to 100 μl and injected into LC-MS/MS. The nucleosides were separated by reverse phase high-performance liquid chromatography on an Agilent C18 column, coupled with mass spectrometry detection using AB SCIEX QTRAP 5500. The m6A levels were calculated as the ratio of m6A to A based on the calibrated concentrations according to the standard curve obtained from pure nucleoside standards running with the same batch of samples.

### Oligonucleotide transfection and lentivirus transduction

Synthetic siRNA oligonucleotides specific for regions in the METTL3, WTAP, FTO, YTHDF2, CTNNB1 mRNA were designed and synthesized by GenePharma (Shanghai, China). The sequences that gave successful knockdown were generalized in Additional file 5: Table S3. Cells were transfected with the oligonucleotides using Lipofectamine 20,000 (Invitrogen) as per the manufacturer’s instructions. To construct METTL3 stable knockdown cell lines the shRNA against METTL3 and the control shRNA obtained from General Biosystems (Anhui, China) were used. Lentivirus were packaged in HEK293T cells through co-transfecting every shRNA construct with packing vectors (PsPAX2, pMD2.G) into HEK293T cells, the lentivirus particles were harvested at 24 h, 48 h and 72 h and directly infected HepG2 and Huh6 cells under polybrene (Santa Cruz Biotechnology) for 12–24 h. Then, positively transfected HepG2 and Huh6 cells were selected for 7–10 days using 2 μg/ml puromycin (Invitrogen).

### Animal studies

Animal studies were approved by the Institutional Animal Care and Use Committee of Shanghai Children’s Medical Center, Shanghai, China. Four-week-old male nude mice (*n* = 7) purchased from Shanghai Super-B&K Laboratory Animal Crop (Shanghai, China) were used. 5 × 10^6^ cells were subcutaneously injected into the left or right flank of each mouse. Tumor volume was measured every 2 days and calculated using the following formula: Volume (mm^3^) = Length (mm) × Width^2 (^mm^2)^ /2. Mice were sacrificed after 26 days and tumor weighed was noted.

### RNA stability assays

To detect the lifetime of CTNNB1, cells were incubated with actinomycin (5 mg/ml) to terminate the transcription. Samples were collected at 0, 30, 60, 120 min post-termination. The total RNA was extracted and the remaining CTNNB1 was determined by RT-qPCR.

### Statistics

All the data were presented as mean ± standard deviation. Differences between two groups or multiple groups were analyzed by Student’s t-test and ANOVA, respectively. Pearson’s X^2^ test was used to examine the relationships between METTL3 expression levels and clinicopathological characteristics. Kaplan-Meier analysis and log-rank test were used to evaluate the differences in patient survival. The receiver operating characteristic (ROC) curves were generated to evaluate the diagnostic value. Statistical analyses were performed using the SPSS software package ver20.0 (SPSS, Inc., Chicago, IL, USA) and GraphPad Prism 7.0 (GraphPad Software, La Jolla, CA, USA). Data were considered statistically significant as follows: *p-value< 0.05, **p-value< 0.01, ***p-value< 0.001 and ****p-value< 0.0001.

## Results

### The abnormal m^6^A modification in HB and the functional roles of METTL3, WTAP, FTO, YTHDF2 in HB cells

**Fig. 1:**
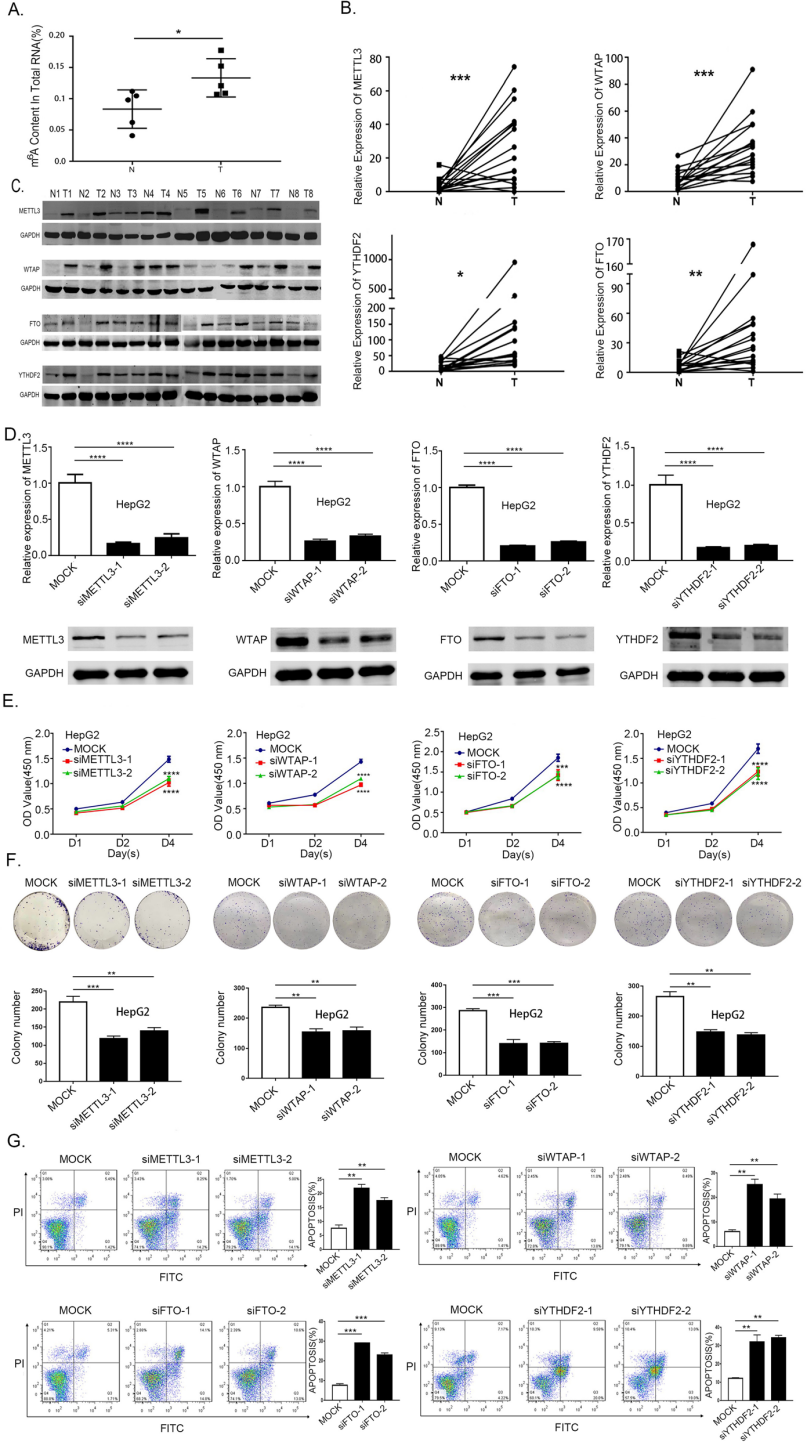
Frequent up-regulation of m^6^A methylation in HB and m6A factors affected HB cell proliferation and apoptosis. **a** Elevation of m^6^A mRNA methylation level in HB tumor and normal samples determined by m^6^A enzyme-linked immunosorbent assay (paired samples t-test). **b** mRNA expression of m^6^A regulators in 16 pairs of HB determined by RT-qPCR (paired samples t-test). **c** Protein expression of m^6^A regulators validated in 8 pairs of HB and nontumor samples by western blotting. **d** HepG2 cells were transfected with siMETTL3, siWTAP, siFTO, siYTHDF2 and the knockdown effect was verified at mRNA and protein levels (one-way analysis of variance, Dunnett’s test). **e** HepG2 cells were transfected with siMETTL3, siWTAP, siFTO, siYTHDF2 followed by CCK8 assays. (one-way analysis of variance, Dunnett’s test). **f** Knockdown of METTL3, WTAP, FTO, YTHDF2 in HepG2 cells subjected to colony formation assays, histogram of colony formation assays from 3 independent experiments (one-way analysis of variance, Dunnett’s test). **g** Flow cytometry assays used to evaluate the apoptosis in HepG2 cells transfected with siMETTL3, siWTAP, siFTO, siYTHDF2, histogram of flow cytometry assays from 3 independent experiments (one-way analysis of variance, Dunnett’s test). Abbreviations: N, normal; T, tumor. *p-value< 0.05, **p-value< 0.01, ***p-value< 0.001, ****p-value< 0.0001

To explore the potential role of m^6^A modification in HB, we first examined m^6^A levels in the tumor and normal tissues. The elevation in the m^6^A mRNA level was identified in tumor tissues (Fig. 1a) and HB cells (Additional file 1: Figure S1A). Additionally, we also evaluated the expression of m^6^A writers, erasers and readers in tumor and adjacent normal hepatic tissues by quantitative PCR coupled with reverse transcription (RT-qPCR) and Western blotting. We found that most of the tumors exhibit significantly up-regulated METTL3, WTAP, FTO and YTHDF2 levels when compared against adjacent normal tissues (Fig. 1b and c). In contrast, no significant difference was observed in the expression of METTL14, KIAA1429 and ALKBH5 between the tumor and normal tissues (Additional file 1: Figure S1B). Overall, these results confirmed that m^6^A modification was indeed up-regulated in tumor tissue.

To investigate the functional roles of METTL3, WTAP, FTO, YTHDF2 in HB. We established METTL3, WTAP, FTO, YTHDF2 knockdown HepG2 cells with two independent siRNA sequences. RT-qPCR and Western blotting were used to investigate the successful knockdown of METTL3, WTAP, FTO, YTHDF2 in HepG2 cells (Fig. 1d). The knockdown of METTL3, WTAP, FTO, YTHDF2 genes remarkably suppressed HB cell proliferation (Fig. 1e) and inhibited their colony-forming ability (Fig. 1f). Furthermore, annexin V-PI double staining assay revealed that METTL3, WTAP, FTO, YTHDF2 knockdown can also induce apoptosis (Fig. 1g). Considering the catalytic function of these genes and the up-regulated level of m^6^A modification. We finally choose METTL3 as the candidate molecule as a marker for the aberrant m^6^A modification in HB. It may act as an oncogene promoting HB proliferation and can also serve as a crucial active component of the m^6^A methyltransferase having a positive correlation with m^6^A levels.

### METTL3 as a potential diagnostic and prognostic biomarker for HB patients

**Fig. 2:**
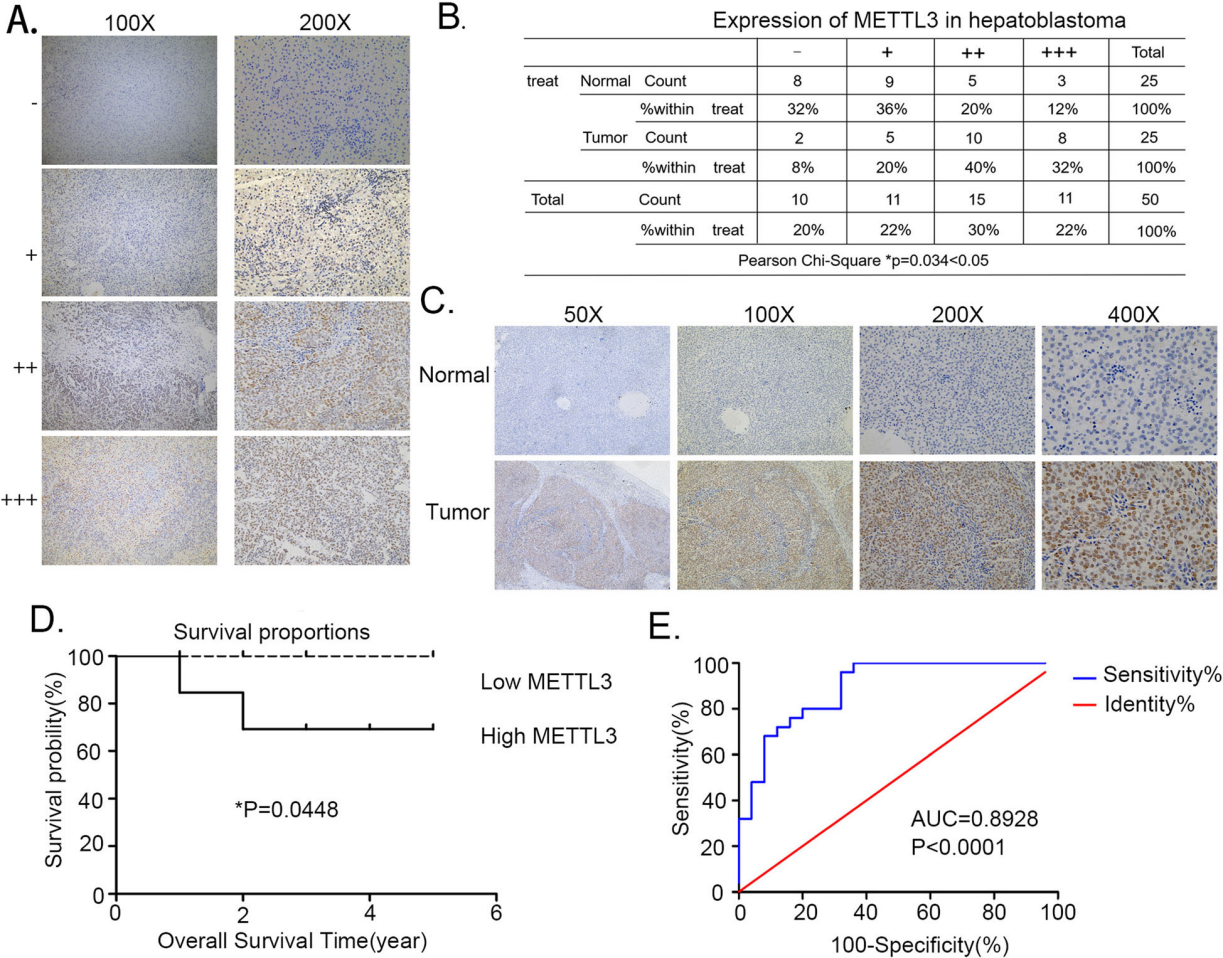
METTL3 serves as a prognostic factor in HB. **a** The METTL3 expression level was defined by grading criteria based upon the percentage of cells with differential IHC staining intensities in 25 pairs of HB tissues. **b** The Pearson’s X^2^ test of METTL3 in 25 paired HB tissues. **c** Representative IHC stains of METTL3 in matched HB tissues. **d** Kaplan-Meier survival curves of OS in 25 HB patients based on METTL3 IHC stains. Patients were assigned to two subgroups according to the median METTL3 IHC stain score (log-rank test). **e** ROC curve for using METTL3 as a diagnostic biomarker. *p-value< 0.05, ****p-value<0.0001

As m^6^A modification exhibits an increasing tend with HB (Fig. 1a), a possible link between METTL3 and HB development was evaluated. Immunohistochemistry on 25 pairs of HB patient samples was performed. Based upon the grading criteria (Fig. 2a) and the Pearson’s X^2^ tests (Fig. 2b), findings revealed an increase in METTL3 levels in HB (Fig. 2c). There were consistent with our previous observations at the mRNA and protein levels (Fig. 1b, Fig. 1c). In the next step, patients’ clinical data was preprocessed to explore the relationship between METTL3 expression and their clinicopathological characteristics. RT-qPCR method was used to detected the METTL3 expression in tissue samples. Based upon the median ΔCT value it was divided into high and low group. Kaplan Meier analysis revealed that patients with increased METTL3 expression had frequent recurrence and had poor survival (Fig. 2d). ROC curve further confirmed the diagnostic value of METTL3 (AUC = 0.8928; *P*-value< 0.0001) (Fig. 2e). Taken together, these results suggest that METTL3 and could serve as a promising biomarker for the diagnosis and prognosis of HB patients.

### METTL3 promotes HB tumor growth in vivo

**Fig. 3:**
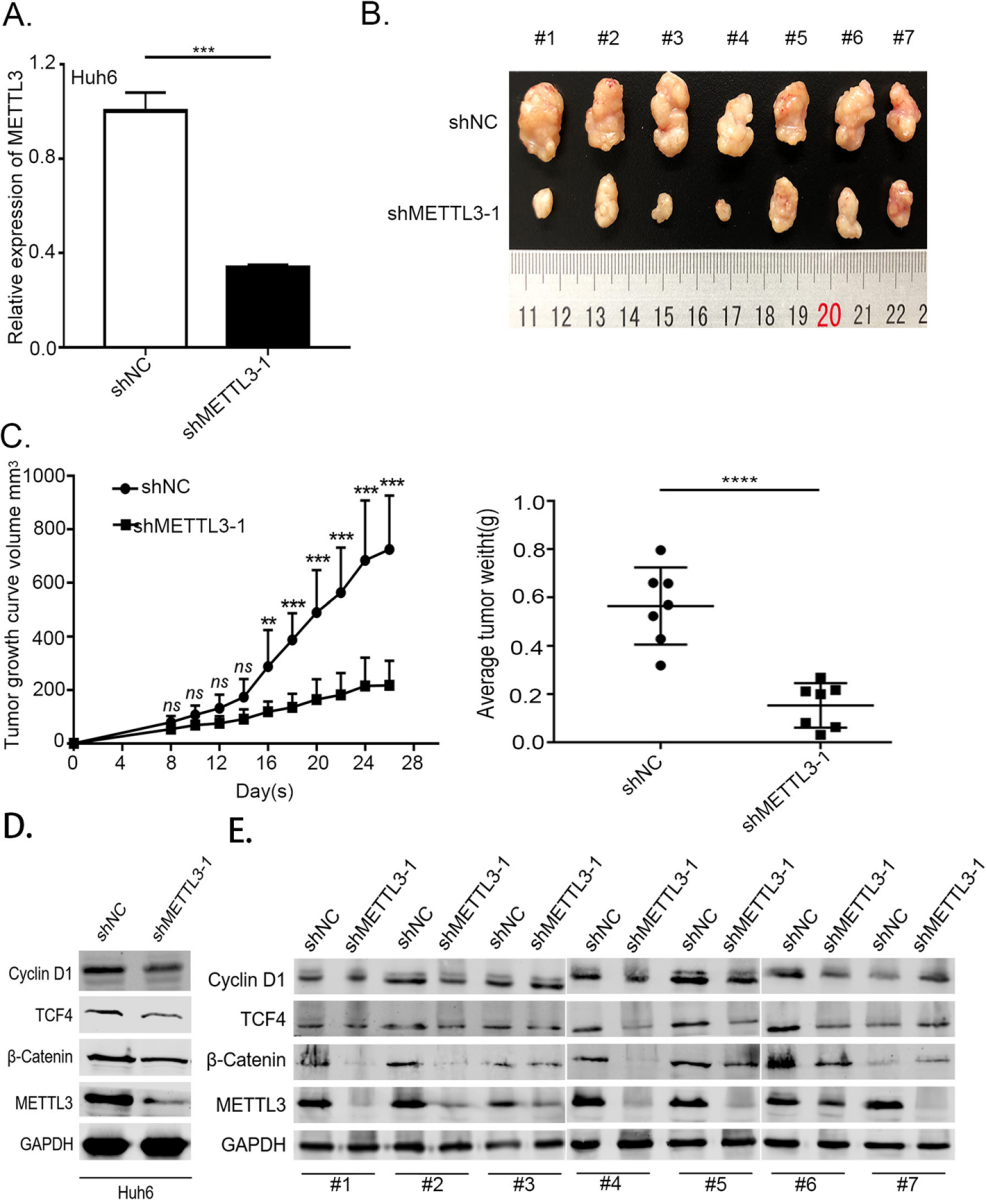
Silencing METTL3 inhibited HB tumor growth in nude mice models. **a** RT-qPCR was used to verify the METTL3 knockdown efficiency using shRNA in Huh6 cells. (independent-samples t-test). **b** Knockdown of METTL3 effectively suppressed the HB subcutaneous tumor growth in nude mice. **c** Tumor size and weight at the endpoint. Tumor size was monitored every 4th days. (two-way ANOVA followed by Bonferroni’s test and independent-samples t-test). **d** Western blotting was used to detect the expression of TCF4 and Cyclin D1 in METTL3 knockout HB cell Huh6. **e** Western blotting was used to detect the expression of TCF4 and Cyclin D1 in mice tumor-bearing tissue ns, no significant difference. *p-value< 0.05,**p-value< 0.01, ***p-value< 0.001, ****p-value< 0.0001

Further to verify the oncogenic potential of METTL3 in relation with HB, we performed a subcutaneous implantation experiment in nude mice to test the effect of METTL3 knockdown in HB tumorigenicity. We used the Huh6 cells infected with lentivirus expressing shRNA against METTL3 (shMETTL3–1) and its negative control (shNC). The RT-qPCR analysis confirmed that METTL3 expression was significantly downregulated in Huh6 cells infected with shMETTL3–1 compared to the negative control (Fig. 3a). From the in vivo experiment, we observed that stable knockdown of METTL3 can cause significant reduction in tumor size and weight with respect to the nontarget shRNA control (Fig. 3b and c). Further more, we confirmed decreased expression of CTNNB1 downstream transcription factor TCF4 and target gene Cyclin D1 in METTL3 knockout HB cell Huh6 and tumor-bearing tissues of mice (Fig. 3d and e). Collectively, these experiments support the oncogenic role of METTL3 in HB tumorigenesis and development.

### Widespread m^6^A modification of HB and identifies important biological pathways with altered methylation in HB tumors

**Fig. 4:**
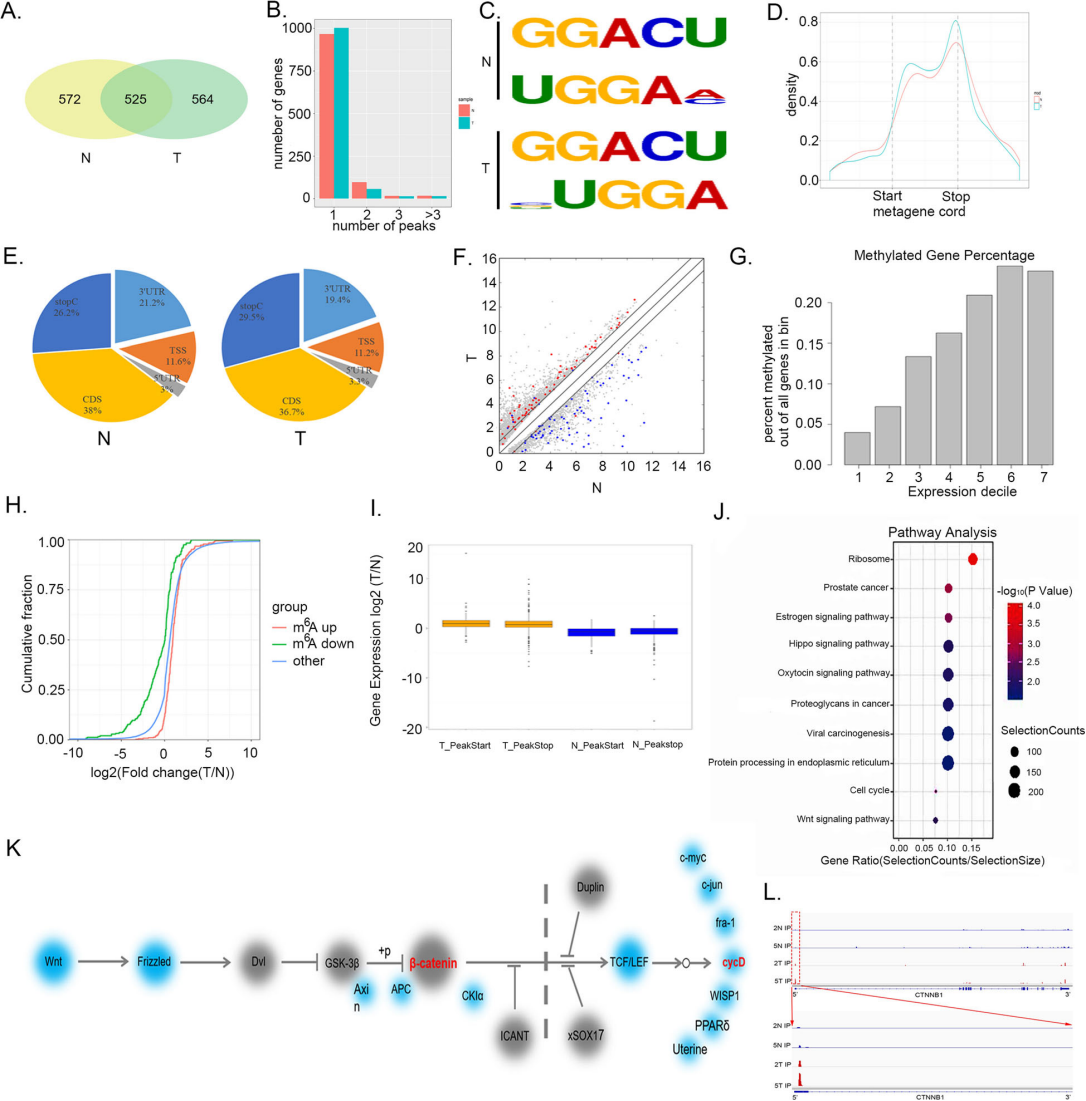
m^6^A-Seq profiling of HB and identified CTNNB1 as a target of METTL3-mediated m^6^A modification. **a** Venn diagram of m^6^A modified genes in tumor and normal tissues. **b** The relationship between the number of genes and the peaks. **c** Top consensus motif identified DREME with m^6^A-seq in HB and normal tissues. **d** The normalized distribution of m^6^A peaks across the start codon, CDS, stop codon of mRNAs for normal and tumor m^6^A peaks. **e** Graphs of m^6^A peak distribution showing the proportion of total m^6^A peaks in the indicated regions in normal and tumor. **f** Comparison of the abundance of high level m^6^A peaks across the transcriptome of normal and tumor tissues. Genes with normal specific m^6^A peaks are highlighted in blue and genes with tumor-specific m^6^A peaks are highlighted in red. **g** Bar figure for the abundance of m^6^A modification percentage and gene expression. **h** Cumulative distribution curve for the gene expression changes between the tumor and normal tissues for up-regulated m^6^A methylation (Red) and down-regulated m^6^A methylation (Green). **i** The ratio of genes expression in normal (N) and tumor (T) tissues containing specific m^6^A peaks. Genes according to the peak position are divided into two groups (PeakStart and PeakStop). **j** KEGG pathway analysis of transcripts with increased m^6^A methylation and up-regulated mRNA expression in tumors against normal tissues. **k** Diagram of the Wnt/β-catenin pathway with genes affected by m^6^A marked by red. The diagram is based on KEGG annotations. **l** The m^6^A abundances on CTNNB1 mRNA transcripts in HB normal tissues and tumor tissues as detected by m^6^A-seq

In order to obtain a transcriptome-wide m^6^A map in HB, we performed m^6^A-seq analysis of five tumor and normal tissues. All five tumors exhibited high amount of total m^6^A levels. m^6^A-seq identified 4071 peaks in 1089 genes in tumor tissues. Among the 1089 genes, 525genes were common between the normal and tumor tissues (Fig. 4a). We also analyzed a possible relationship between m^6^A peaks and gene number in the normal and tumor tissues (Fig. 4b). To determine if the m^6^A peaks had consensus sequence of RRACH (where RR Represents purine, A is m^6^A and H is a non-guanine base), the top 1000 most significant peaks were analyzed,we found at least two motifs among the significant peaks representing the most common consensus in normal tissue and tumor tissue (Fig. 4c). We further investigated the distribution pattern of m^6^A across the whole transcriptome for both normal and tumor tissues. A similar pattern of m^6^A distribution was observed when the RNA species were divided into stop codon, coding sequence (CDS), start codon regions of mRNAs and non-coding RNAs. Our findings suggest that the reads from m^6^A-IP are highly enriched around the stop codon and CDS in both normal and tumor tissues (Fig. 4d). Further to confirm the preferential locations of m^6^A on transcripts, we investigated the metagene profiles of m^6^A peaks. Consistent with the distribution of reads, m^6^A peaks were abundant near the CDS (38%) and stop codon (26.2%) followed by the 3’UTR (19.4%) and then start codon (11.2%) in normal tissues. In tumor, the m^6^A peaks were abundant near the CDS (36.7%) and stop codon (29.5%) followed by the 3’UTR (19.4%) and start codon (11.2%) (Fig. 4e).

Further to explore the relationship between m^6^A peaks and mRNA expression differentially expressed genes in normal and tumor tissues from the m^6^A-Seq data were identified. There were 1440 strongly expressed genes in normal as compared to 2868 genes in tumor. Specific genes containing high level m^6^A peaks in normal were identified and labeled in blue. Similarly, in tumor these were marked in red color (Fig. 4f). Furthermore, we also observed a linear relationship between expression and percentage methylation of all genes in the bin (Fig. 4g). We extended our analysis to the whole transcriptome. The findings suggest that m^6^A modification leads to more genes upregulation in the tumor as compared to normal (Fig. 4h). The positions of m^6^A peaks involved in upregulated genes were also evaluated and it was observed that a large amount of these peaks were located at the 5′ end of the corresponding genes. We separated m^6^A-containing genes into PeakStart and PeakStop groups and checked how the genes expression is related to the locations of m^6^A peaks. Findings indicate that genes in the PeakStart and PeakStop category have overall higher expression levels and correlate well with m^6^A peaks in tumor (Fig. 4i).

In order to determine the effect of m^6^A in HB, we selected strongly expressed genes with up-regulated m^6^A methylation and identified the KEGG pathway. The KEGG pathway analyses were performed to organize and identify differentially activated biological processes between normal and tumor tissues. The significant genes in the tumor were related with viral carcinogenesis, proteoglycans in cancer, protein processing in endoplasmic reticulum and Wnt signaling pathway and so on. As HB is a Wnt/β-catenin-driven malignancy, we hypothesized that up-regulated m^6^A methylation might promote tumor growth through activation of the Wnt/β-catenin signaling pathway. Therefore, Wnt/β-catenin signaling pathway was chosen for further research (Fig. 4j). Indeed, several genes such as CTNNB1, CCND1 and NKD1 involved in the Wnt/β-catenin signaling pathway showed increased m^6^A methylation in tumor tissues with respect to normal (Fig. 4k). Figure 4l showed the m^6^A abundances of CTNNB1 were markedly increased HB tumor tissues. Overall these results are supportive of the fact that m^6^A has tendency of demonstrating a positive correlation with gene expression in large fraction of HB transcripts and activate m^6^A methylation activates the Wnt/β-catenin signaling pathway.

### m^6^A methylation regulates activation of CTNNB1

**Fig. 5:**
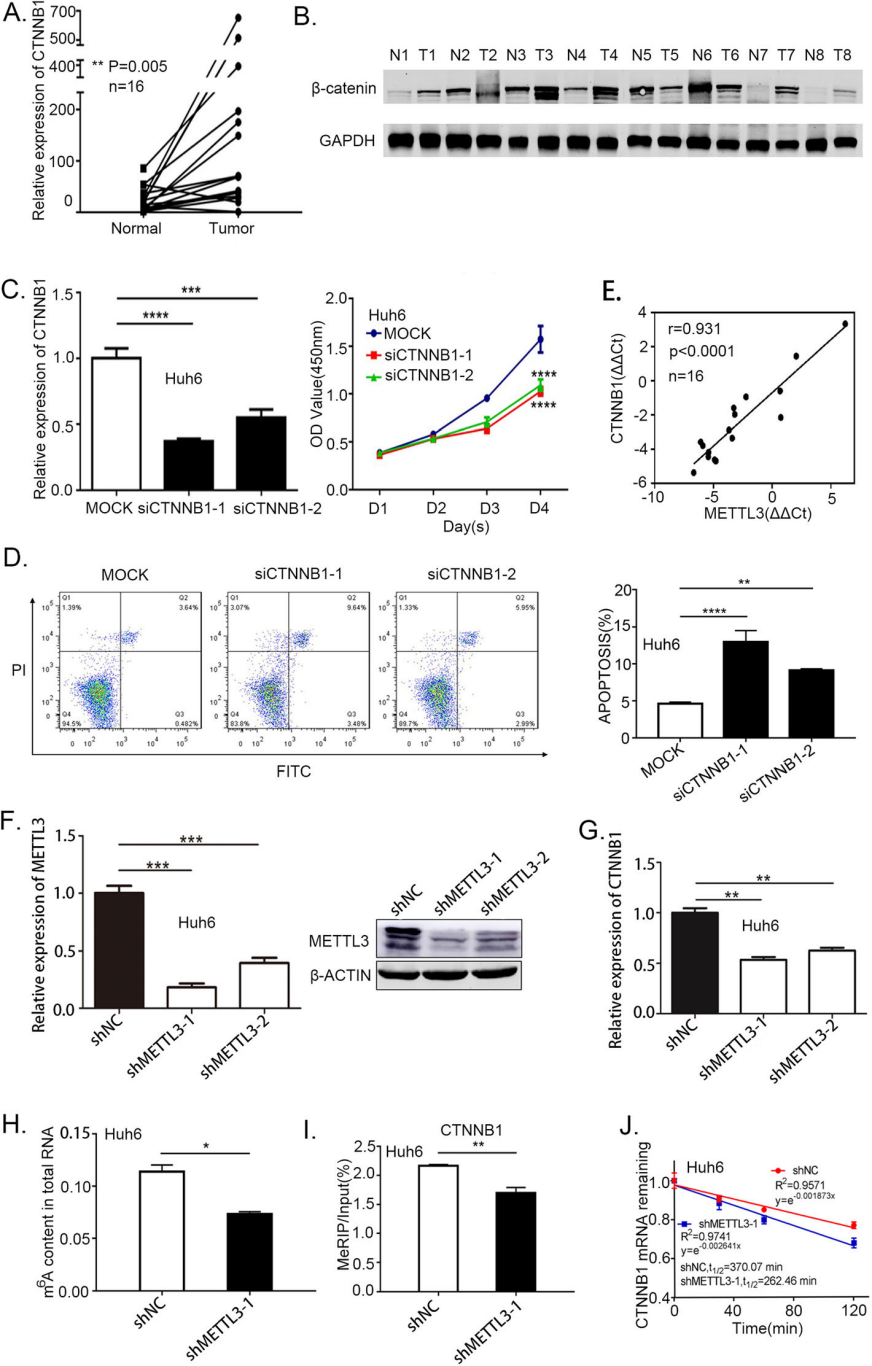
Reduced m^6^A methylation reduced CTNNB1 expression and stability. **a-b** The relative mRNA expression and protein expression of CTNNB1/β-catenin in tumor and normal tissues (paired samples t-test). **c** The viability of CTNNB1 knockdown in Huh6 cells measured by CCK8 assay (one-way analysis of variance, Dunnett’s test). **d** The apoptosis rate in CTNNB1 knockdown in Huh6 cells were measured by FACS assays, histogram of flow cytometry assays from 3 independent experiments (one-way analysis of variance, Dunnett’s test) (**e**) Scatter plot showing the correlation between METTL3 and CTNNB1 expression. The linear best fit line is shown. The Pearson correlation coefficient (r) and p-value (P) obtained from two-tailed t-test of *r* = 0.931 are shown *N* = 16 tumor-normal pairs. **f** METTL3 stable knockdown in Huh6 cells were generated by lentiviral-based shRNA expression. METTL3 knockdown efficiency was confirmed at both the mRNA and protein levels by RT-qPCR and western blotting (one-way analysis of variance, Dunnett’s test). **g** CTNNB1 was quantified by RT-qPCR upon METTL3 depletion in Huh6 cells (one-way analysis of variance, Dunnett’s test). **h** Relative m^6^A level in knockdown METTL3 in Huh6 cells (independent-samples t-test). **i** m^6^A-IP combined with RT-qPCR was used to quantify the relative m^6^A modified level of CTNNB1 upon METTL3 depletion in Huh6 cells (independent-samples t test). **j** Lifespans of CTNNB1 expression in cells transfected with the reduction of METTL3 in Huh6 cells. Relative mRNA levels were quantified by RT-qPCR. *p-value< 0.05,**p-value< 0.01,***p-value< 0.001, ****p-value< 0.0001

Among CTNNB1, CCND1 and NKD1 we chose CTNNB1 as our candidate gene, as CTNNB1 is a key component of Wnt/β-catenin signaling pathway, and almost 50–90% of HB has the CTNNB1 mutation. We first confirmed CTNNB1 up regulation in HB tumor tissues (Fig. 5a and b). Knockdown CTNNB1 reduced the HB cells viability (Fig. 5c, Additional file 2: Figure S2A) and induced apoptosis (Fig. 5d, Additional file 2: Figure S2B, S2C). Furthermore, we also noticed a significantly positive correlation between CTNNB1 and METTL3 expression levels (Fig. 5e). To evaluate the relationship between METTL3 and CTNNB1, we first confirmed METTL3 was high-regulated in Huh6 and HepG2 cells (Addition file 2: Figure S2D), then we used Huh6 and HepG2 cells infected with lentivirus expressing shRNA against METTL3 (shMETTL3), or its negative control (shNC). Data from RT-qPCR and Western blotting analysis confirmed that METTL3 expression was significantly down-regulated in Huh6 and HepG2 cells infected with shMETTL3 with respect to the negative control cells (Fig. 5f, Additional file 2: Figure S2E). The results also demonstrated that reduced METTL3 expression can decrease the CTNNB1 expression (Fig. 5g, Additional file 2: Figure S2F). We then evaluated if knockdown METTL3 has a negative effect on m^6^A level using a colorimetric quantification assay (Fig. 5h, Additional file 2: Figure S2G). Next, we evaluated CTNNB1 in the METTL3 knockdown cells by m^6^A immunoprecipitation (m^6^A-IP) followed by RT-qPCR, which confirmed the reduction of CTNNB1 m^6^A methylation (Fig. 5i, Additional file 2: Figure S2H). The stability of CTNNB1 expression were evaluated by METTL3 knockdown cells. The results demonstrated that reduced m^6^A methylation can decrease the CTNNB1 stability (Fig. 5j, Addition file 2: Figure S2I). Taken together, the findings indicate that CTNNB1 is a downstream target of METTL3 in HB.

## Discussion

The discovery and mapping of m^6^A demethylases in mammalian systems suggests that m^6^A methylation of mRNA is a reversible and dynamic process with regulatory functions. Through a functional interplay between m^6^A methyltransferase and demethylases, the dynamic m^6^A modification is implicated in a variety of cellular processes, such as RNA splicing, protein translation, and stem cell renewal. Recently developed, antibody-based, high-throughput sequencing technology has allowed researchers to precisely map m^6^A sites to obtain further insights. Although, m^6^A is considered as the most prevalent alteration in human mRNA. Very few studies have focused upon the role of m^6^A modification in HB. In the present study we observed an increased tendency of m^6^A modification in HB, suggesting its potentially involvement in the malignancy. Genes METTL3, WTAP, FTO and YTHDF2 were up-regulated, and they all seem to play an essential role in promoting HB growth. Among the m^6^A related proteins, METTL3 and WTAP were chosen for further research as they were the m^6^A writers and have increased tendency of m^6^A modification. However, WTAP lacks methyltransferase domains and shows no catalytic activity of m^6^A modification. Collectively, it was found that METTL3 was the main factor for the aberrant m^6^A modification in HB.

The m^6^A levels vary in different tissues and cell lines. The loss of m^6^A can promote self-renewal and hinder differentiation of mouse embryonic stem cells [31]. For instance, knockdown of mettl3 had led to reduced self-renewal abilities in mouse embryonic stem cells [32]. However, a study had demonstrated that m^6^A modification was decreased in hepatocellular carcinoma, especially in metastatic hepatocellular carcinoma, and METTL14 was the main factor involved in aberrant m^6^A modification [14]. These studies indicate that m^6^A modification is indeed a complicated regulation network in different tissues.

Previous studies have shown that m^6^A plays a critical role in HB. Our findings also revealed transcriptome-wide m^6^A distributions in HB normal and tumor tissues. We found that m^6^A is highly conserved across normal and tumor tissues. The m^6^A-Seq data suggest that m^6^A distribution in normal and tumor mRNA is enriched around the CDS which is distinct from mammals. Also, both in normal and tumor tissues the enrichment of m^6^A correlates positively with the overall upregulation of mRNA expression level. Nevertheless, there were different methylated mRNA between normal and tumor tissues. Methylation levels of 243 and 200 m^6^A mRNAs were upregulated in tumor tissue and normal tissues. Which provide a starting roadmap for uncovering m^6^A functions in HB proliferation.

The Wnt/β-catenin pathway is frequently mutated and overactive in solid malignancies promoting tumor development. In HB, driving and most recurrently mutated proto-oncogene with 50–90% frequency is CTNNB1. In this study, we discovered that m^6^A mRNA methylation regulates the Wnt/β-catenin pathway significantly. In HB, m^6^A methylation normally induces Wnt/β-catenin pathway activity by promoting the expression of CTNNB1. Using cultured HB cells, we revealed that reduced m^6^A methylation leads to a decrease in CTNNB1 expression and stability. We also used m^6^A experiment to confirm that decreased m^6^A methylation further decreased CTNNB1 m^6^A modification enrichment. Our m^6^A-Seq results from HB tumors and normal tissues along with our mechanistic studies in HB cells indicate that regulation of CTNNB1 m^6^A modification as an important mediator of these changes for cell proliferation. In our future studies we will be exploring the relationship between the m^6^A modification and CTNNB1 mutation. Also the question if m^6^A modification can induce mutated CTNNB1 to accumulate in the nucleus and promote the binding the TCF/LEF transcription factor will be elucidated further.

## Conclusion

In summary, our study delineates a METTL3-m^6^A module that regulates the process of CTNNB1 with respect to its functional implications in HB proliferation. Our results suggest that m^6^A modification plays a critical role in tumor biology. Also, the m^6^A modification of CTNNB1 might be a general growth mechanism affecting a range of other biological processes, that warrants further exploration.

## Supplementary information

**Additional file 1: Figure S1.** The m^6^A mRNA level in HB cells and the expression of METTL14, KIAA1429 and ALKBH5 in HB tumor tissues. (A) LC-MS/MS quantification of the m6A/A ratio in total RNA isolated from hepatocytes and HB cells (one-way analysis of variance, Dunnett’s test). (B) The expression of METTL14, KIAA1429 and ALKBH5 were quantified by RT-qPCR in 10 paired HB tumor and normal tissues (paired samples t-test). ns, no significant difference. **p-value< 0.01,***p-value< 0.001.

**Additional file 2: Figure S2.** Reduced m^6^A methylation decreases CTNNB1 expression and stability in HepG2 cells. (A) CCK8 assay was used to evaluate the viability of CTNNB1 knockdown in HepG2 cells measured (one-way analysis of variance, Dunnett’s test). (B) The apoptosis rate in CTNNB1 knockdown in HepG2 cells were measured by FACS assays. (C) Histogram of flow cytometry assays from 3 independent experiments (one-way analysis of variance, Dunnett’s test). (D) METTL3 expression in hepatocytes and HB cells was evaluated by RT-qPCR (one-way analysis of variance, Dunnett’s test). (E) METTL3 stable knockdown in HepG2 cells were generated by lentiviral-based shRNA expression. METTL3 knockdown efficiency was confirmed at both the mRNA and protein levels by RT-qPCR and western blotting (one-way analysis of variance, Dunnett’s test). (F) RT-qPCR was used to test the expression of CTNNB1 upon METTL3 knockdown in HepG2 cells (one-way analysis of variance, Dunnett’s test). (G) Relative m^6^A level in knockdown METTL3 in HepG2 cells (independent-samples t-test). (H) m^6^A-IP combined with RT-qPCR was used to quantify the relative m^6^A modified level of CTNNB1 upon METTL3 depletion in HepG2 cells (independent-samples t test). (I) Lifespans of CTNNB1 expression in cells transfected with the shMETTL3 in HepG2 cells. Relative mRNA levels were quantified by RT-qPCR. *p-value< 0.05,**p-value< 0.01,***p-value< 0.001, ****p-value<0.0001.

**Additional file 3: Table S1.** Sequence of primers used in this study.

**Additional file 4: Table S2.** Antibodies used in this study.

**Additional file 5: Table S3.** Targets sequences of siRNAs used in this study.

## Data Availability

All data generated or analyzed during the current study are included in this published article (and its supplementary information files).

## Abbreviations

ALKBH5: AlkB homoogue 5
CDS: Coding Sequence
DMEM: Dulbecco’s Modified Eagle’s Medium
DNA: Deoxyribonucleic acid
FBS: Fetal Bovin Serum
FTO: Fat mass and obesity-associatd protein
HB: Hepatoblastoma
m^6^A: N6-methyladenosine
MEM: Minimum Essential Medium
MeRIP: Methylated RNA immunoprecipitation
METTL14: Methyltransferase-like 14
METTL3: Methyltransferase-like 13
mRNA: Messenger RNA
N: Normal
PI: Propidium iodide
PVDF: Polyvinylidene fluoride
RNA-Seq: RNA-sequencing
ROC: Receiver operating characteristic
RT-qPCR: Reverse transcription quantitative real-time PCR
SDS: Sodium dodecyl sulfate
SDS-PAGE: Sodium dodecyl sulfate-polyacrylamide gel electrophoresis
SOCS2: Suppressor Of Cytokine Signaling 2
T: Tumor
WTAP: Wilms tumor 1-assocated protein
YTH: YT521-B homology
ZC3H13zinc: Finger CCCH domain-containing protein 13
