# Correction to: m6A mRNA methylation regulates CTNNB1 to promote the proliferation of hepatoblastoma

**DOI:** 10.1186/s12943-020-1136-6

**Published:** 2020-02-04

**Authors:** Li Liu, Jing Wang, Guifeng Sun, Qiong Wu, Ji Ma, Xin Zhang, Nan Huang, Zhixuan Bian, Song Gu, Min Xu, Minzhi Yin, Fenyong Sun, Qiuhui Pan

**Affiliations:** 1 Department of Clinical Laboratory Medicine, Shanghai Children’s Medical Center, School of medicine, Shanghai Jiaotong University, Shanghai, 200127 China; 2 Department of Surgery, Shanghai Children’s Medical Center, School of medicine, Shanghai Jiaotong University, Shanghai, 200127 China; 3 Department of Clinical Laboratory Medicine, Shanghai Tenth People’s Hospital of Tongji University, Shanghai, 200072 China; 4 Department of Clinical Laboratory, Shanghai Fourth People’s Hospital affiliated to Tongji University School of Medicine, Shanghai, 200081 China; 5 School of Pharmacy, Guangdong Pharmaceutical University, Gaungzhou, 510006 China; 6 Department of Pathology, Shanghai Children’s Medical Center, School of medicine, Shanghai Jiaotong University, Shanghai, 200127 China

**Correction to: Mol Cancer (2019) 18:188**

**https://doi.org/10.1186/s12943-019-1119-7**

After the publication of this work [1], the authors noticed that the affiliations were incorrectly provided. Updated affiliation section is provided in this paper.
