# Supplementary figures and images for: m6A mRNA methylation regulates CTNNB1 to promote the proliferation of hepatoblastoma
Source: Mol Cancer. 2019 Dec 23;18:188. doi: 10.1186/s12943-019-1119-7 (PMC6927193; doi:10.1186/s12943-019-1119-7)

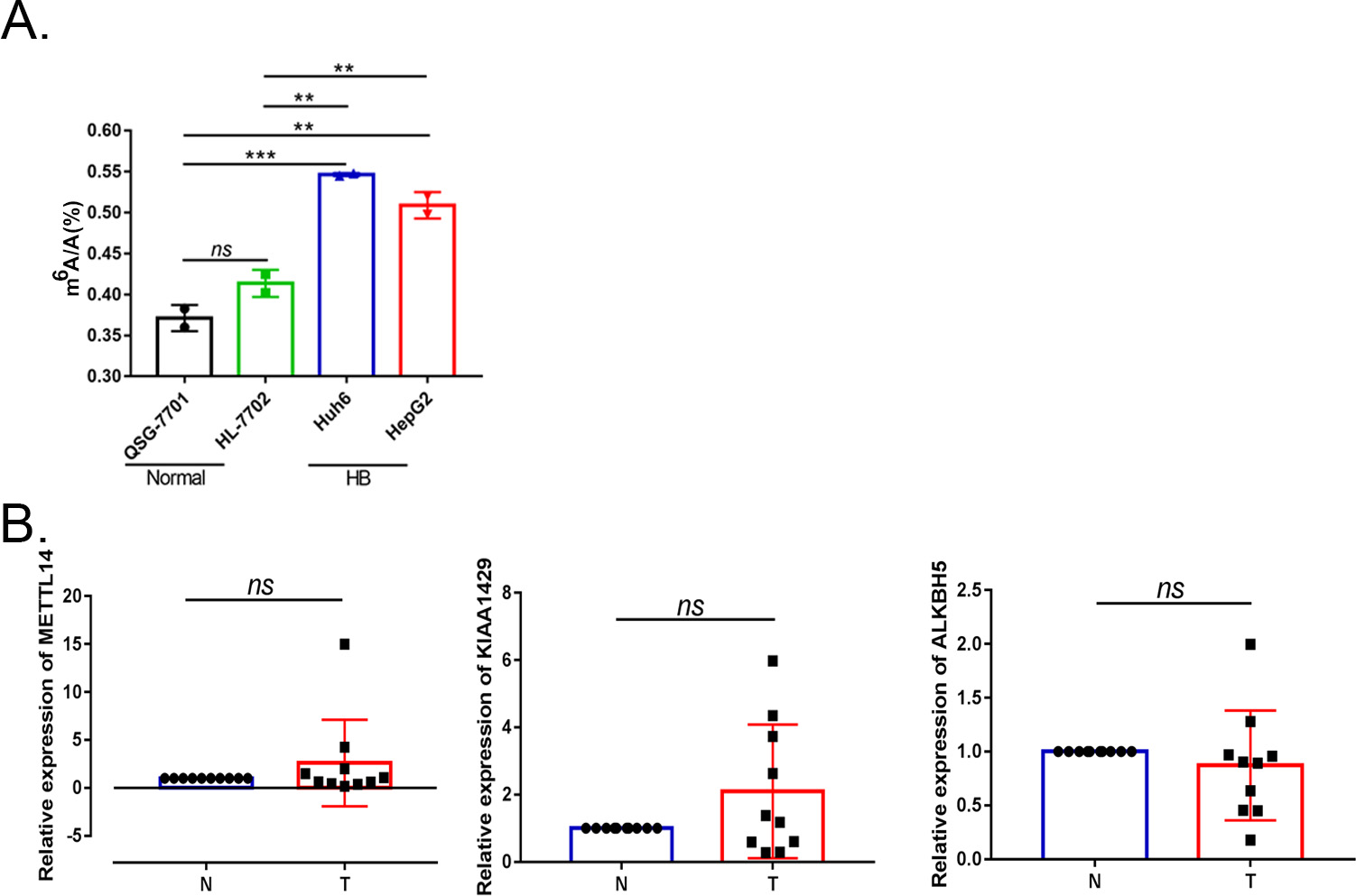

Supplement: Supplementary file 1 — Additional file 1: Figure S1. The m6A mRNA level in HB cells and the expression of METTL14, KIAA1429 and ALKBH5 in HB tumor tissues. (A) LC-MS/MS quantification of the m6A/A ratio in total RNA isolated from hepatocytes and HB cells (one-way analysis of variance, Dunnett’s test). (B) The expression of METTL14, KIAA1429 and ALKBH5 were quantified by RT-qPCR in 10 paired HB tumor and normal tissues (paired samples t-test). ns, no significant difference. **p-value< 0.01,***p-value< 0.001. [file 12943_2019_1119_MOESM1_ESM.jpg]

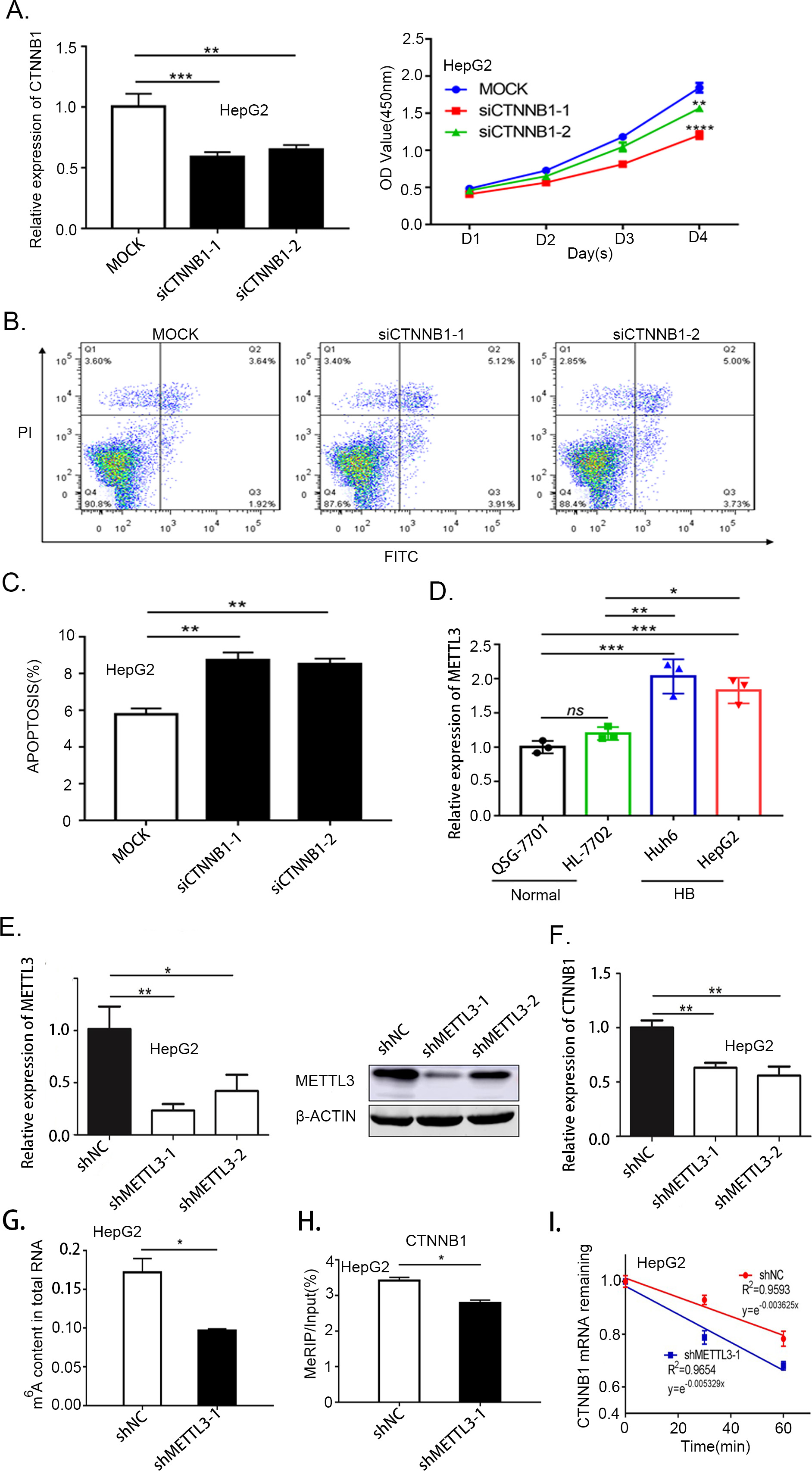

Supplement: Supplementary file 2 — Additional file 2: Figure S2. Reduced m6A methylation decreases CTNNB1 expression and stability in HepG2 cells. (A) CCK8 assay was used to evaluate the viability of CTNNB1 knockdown in HepG2 cells measured (one-way analysis of variance, Dunnett’s test). (B) The apoptosis rate in CTNNB1 knockdown in HepG2 cells were measured by FACS assays. (C) Histogram of flow cytometry assays from 3 independent experiments (one-way analysis of variance, Dunnett’s test). (D) METTL3 expression in hepatocytes and HB cells was evaluated by RT-qPCR (one-way analysis of variance, Dunnett’s test). (E) METTL3 stable knockdown in HepG2 cells were generated by lentiviral-based shRNA expression. METTL3 knockdown efficiency was confirmed at both the mRNA and protein levels by RT-qPCR and western blotting (one-way analysis of variance, Dunnett’s test). (F) RT-qPCR was used to test the expression of CTNNB1 upon METTL3 knockdown in HepG2 cells (one-way analysis of variance, Dunnett’s test). (G) Relative m6A level in knockdown METTL3 in HepG2 cells (independent-samples t-test). (H) m6A-IP combined with RT-qPCR was used to quantify the relative m6A modified level of CTNNB1 upon METTL3 depletion in HepG2 cells (independent-samples t test). (I) Lifespans of CTNNB1 expression in cells transfected with the shMETTL3 in HepG2 cells. Relative mRNA levels were quantified by RT-qPCR. *p-value< 0.05,**p-value< 0.01,***p-value< 0.001, ****p-value<0.0001. [file 12943_2019_1119_MOESM2_ESM.jpg]
